# Highly Elastic Super-Macroporous Cryogels Fabricated by Thermally Induced Crosslinking of 2-Hydroxyethylcellulose with Citric Acid in Solid State

**DOI:** 10.3390/molecules26216370

**Published:** 2021-10-21

**Authors:** Nadegda Bozova, Petar D. Petrov

**Affiliations:** Institute of Polymers, Bulgarian Academy of Sciences, Akad. G. Bonchev st. 103A, 1113 Sofia, Bulgaria; nbozova@polymer.bas.bg

**Keywords:** cryogels, 2-hydroxyethylcellulose, citric acid, green materials

## Abstract

Biopolymer materials have been considered a “green” alternative to petroleum-based polymeric materials. Biopolymers cannot completely replace synthetic polymers, but their application should be extended as much as possible, exploiting the benefits of their low toxicity and biodegradability. This contribution describes a novel strategy for the synthesis of super-macroporous 2-hydroxyethylcellulose (HEC) cryogels. The method involves cryogenic treatment of an aqueous solution of HEC and citric acid (CA), freeze drying, and thermally induced crosslinking of HEC macrochains by CA in a solid state. The effect of reaction temperature (70–180 °C) and CA concentration (5–20 mass % to HEC) on the reaction efficacy and physico-mechanical properties of materials was investigated. Highly elastic cryogels were fabricated, with crosslinking carried out at ≥100 °C. The storage modulus of the newly obtained HEC cryogels was ca. 20 times higher than the modulus of pure HEC cryogels prepared by photochemical crosslinking. HEC cryogels possess an open porous structure, as confirmed by scanning electron microscopy (SEM), and uptake a relatively large amount of water. The swelling degree varied between 17 and 40, depending on the experimental conditions. The degradability of HEC cryogels was demonstrated by acid hydrolysis experiments.

## 1. Introduction

Natural polymers, also referred to as biopolymers, have attracted considerable attention in the last decade as a “green” alternative to petroleum-based polymeric materials [1]. The huge amount of waste from non-degradable synthetic plastics that pollute nature is the main reason to look for environmentally friendly materials and technologies. Biopolymers cannot completely replace synthetic polymers, but their application must be extended as much as possible, exploiting the benefits of their low toxicity and biodegradability. In many cases, however, the weak physical and mechanical properties of biomaterials limit their use. Early successes in fabricating polymer biomaterials obtained from renewable resources have led to commercial products that are mostly used in packaging and as fibers [2]. Polymer biomaterials have been further used for developing products in high-value areas such as flexible electronics, medicine, pharmacology, biotechnology, etc. [3].

Cellulose and its derivatives are among the most abundant natural polymers, and much progress has been made towards their study, modification, and characterization [4]. Cellulose and cellulose derivatives are readily available, biodegradable, nontoxic, and low-cost polymers, and they are widely used for a variety of applications [5]. Cellulose derivatives, unlike pure cellulose, are water-soluble, which makes them an attractive choice for applications related to the biomedicine and bioanalysis fields [6]. Materials based on cellulose derivatives have been evaluated as films and membranes for osseointegration, hemodialysis, biosensors, smart textile fibers, tissue engineering scaffolds, hydrogels, and nanoparticles for drug delivery.

Hydrogels are extremely suitable for use in the pharmaceutical and medical industries. Since hydrogels can retain large amounts of water and because of their soft and elastic consistency, they closely resemble living tissues [7]. In fact, the suitability of hydrogels as biomaterial and their performance in particular applications depends, to a large extent, on the network structure, porosity, and pore size of the hydrogel. Presently, super-macroporous cellulose-based cryogels attract more interest for biomedical applications because of their interconnected super-macropores, with sizes ranging from several to hundreds of microns, which allow the unhindered diffusion of solutes or even colloidal particles [8]. Cryogels are super-macroporous hydrogels synthesized by applying cryogenic treatment of gel-forming systems [9]. For instance, this system could be an aqueous solution of monomer or polymeric precursor and an initiator. The cooling to subzero temperatures leads to phase separation, with the formation of ice crystals and the development of an interconnected porous structure and polymer network. 

Cryogels of cellulose derivatives were prepared by UV-induced crosslinking or reactions with chemical reagents. El-Naggar et al. prepared cryogels loaded with different ratios of silver@titanium oxide nanoparticles by dissolving 2-hydroxyethyl cellulose (HEC) and bacterial cellulose (BC) in water, followed by crosslinking with glyoxal and freeze drying. [10]. The addition of high concentrations of nanoparticles was needed to afford the HEC/BC cryogel with improved mechanical properties compared to the pure HEC/BC cryogel. Macroporous cellulose-based cryogels were fabricated by grafting acrylic acid and acrylamide to cellulose using a cryopolymerization technique [8]. The interconnected macroporous structures of the cellulose-based cryogels were strongly influenced by the amount of crosslinker and extra water added in the process of cryopolymerization. However, the use of toxic chemical reagents often requires additional purification steps to achieve the necessary purity of biomaterials. The crosslinking of cellulose derivatives via UV irradiation of the frozen aqueous solutions of the polymers has been considered a convenient method for fabricating biodegradable cryogels [11]. Moreover, the use of H_2_O_2_ as an initiator produced intact cryogels, which can be used without any purification [12]. The cryogels based on cellulose derivatives were soft and tear-resistant upon gentle handling, but their elastic modulus was notably lower than the elastic modulus of cryogels of synthetic polymers obtained via the same method [13]. Therefore, crosslinking agents (poly(ethylene glycol) diacrylate, bis-acrylamide) and/or chitosan were incorporated into the polymer network to improve the elastic properties of the cryogels [11,14].

To improve the safety of both the final product and the manufacturing process, non-toxic, water-soluble citric acid (CA) was used to crosslink cellulose derivatives in aqueous media [15]. Thus, carboxymethyl cellulose (CMC)/HEC hydrogel and HEC hydrogel were synthesized by heating (60–80 °C) the polymer solution for several hours. It was found that under heating, CA dehydrates to form an intramolecular cyclic anhydride, which reacts with hydroxyl groups in the polymer chains. The remaining CA carboxylic groups dehydrate consecutively to form other intramolecular anhydrides, which react with other hydroxyl groups. Although the crosslinking occurs under the given conditions, the reaction efficiency seems rather low, as the reported gel fraction of the HEC hydrogel was 60% [16]. Since low crosslinking efficiency often leads to weak gels, improving this characteristic is an important prerequisite to fabricate strong materials. Taking into consideration the fact that one of the possible applications of HEC cryogels can be as matrices for the immobilization of enzymes [17] and cells [18], which requires prolonged or repeated use, the mechanical strength of the material is essential. 

In this paper, we report a novel strategy for the synthesis of super-macroporous HEC cryogels with superior elastic properties. Highly efficient crosslinking of HEC with CA was achieved when the reaction was carried out in the absence of any solvent. First, an aqueous solution of HEC and CA was frozen at −20 °C and freeze dried. Then, HEC macrochains were crosslinked by CA in a solid state at an elevated temperature. The effect of reaction temperature and time, as well as CA concentration, on the crosslinking efficacy and physico-mechanical properties of cryogels was investigated in detail. The degradability of the obtained HEC cryogels was demonstrated by acid hydrolysis experiments.

## 2. Results

### 2.1. Synthesis of Cryogels

**Figure 1:**
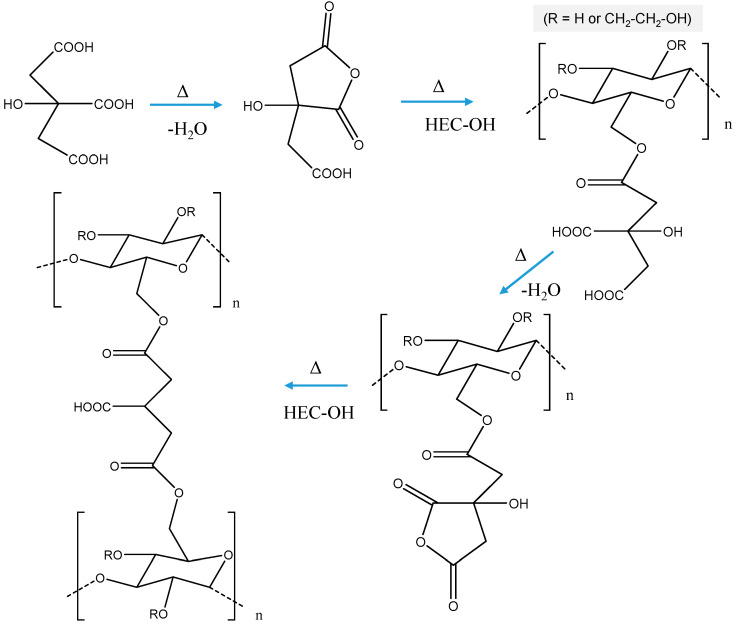
Mechanism of thermally induced crosslinking of 2-hydroxyethylcellulose with citric acid.

HEC cryogels were fabricated by crosslinking high-molar mass HEC with CA at an elevated temperature in the absence of any solvent. A polymer network was formed by successive reactions of CA-based anhydride intermediates with HEC hydroxyl groups (Figure 1).

**Figure 2:**
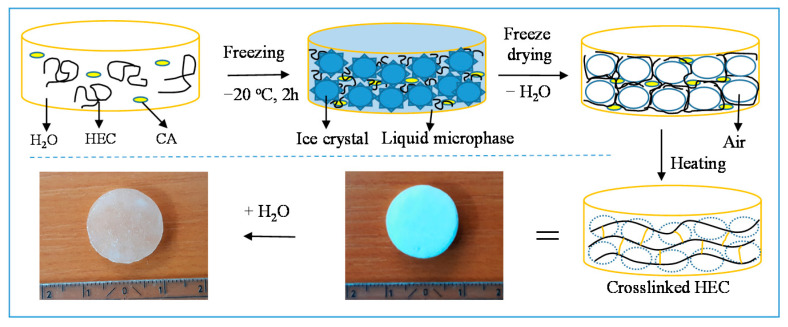
Preparation of cryogel by thermally induced crosslinking of 2-hydroxyethylcellulose with citric acid.

In the first step, an aqueous solution of HEC (2 mass %), containing a given amount of CA (5–20 mass % to the polymer), was divided into portions of 1 mL, frozen, and kept for 2 h at −20 °C. Next, the aqueous phase was removed by lyophilization, and the cryostructured samples were heated in an air atmosphere at high temperatures (70–180 °C) for up to 60 min. Finally, the crosslinked super-macroporous product was placed in water to afford a cryogel (Figure 2).

### 2.2. Influence of CA Concentration, Reaction Temperature, and Reaction Time on the Gel Fraction Yield

**Figure 3:**
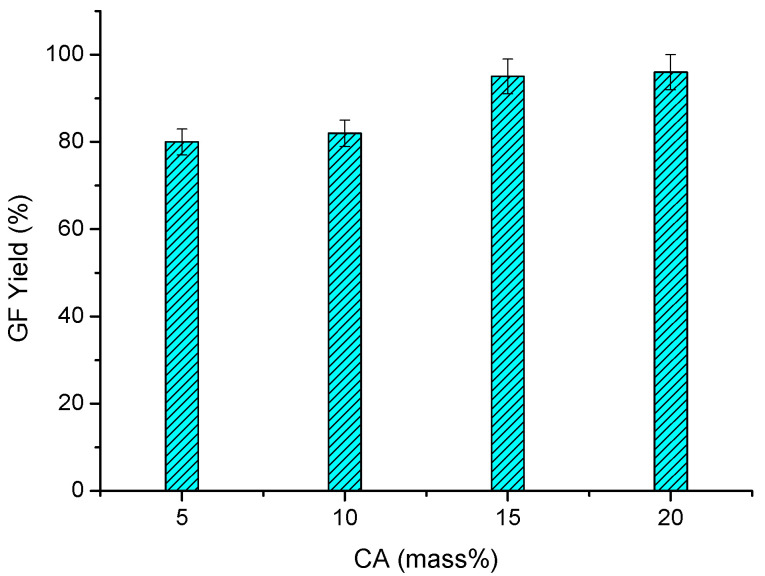
Gel fraction yield of HEC cryogels synthesized with different concentrations of CA. Reaction time = 10 min; temperature = 150 °C.

**Figure A1:**
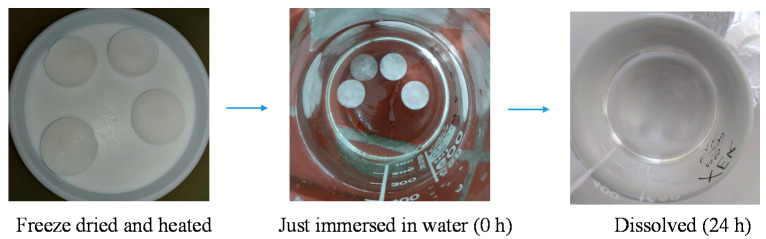
Dissolution in water of HEC samples freeze dried and heated to 150 °C, prepared without CA.

Firstly, the effect of the HEC/CA ratio on the GF yield was studied. The concentration of HEC was kept constant (2 mass %), while the CA content was 5, 10, 15, and 20%, with respect to the polymer mass (Figure 3). The crosslinking reaction was carried out at 150 °C for 10 min. HEC cryogels of good quality (that maintain their structural integrity in water) were obtained even at the lowest CA amount. Besides this, an increase in the GF yield from 80 to 96%, with the rise in CA content from 5 to 20%, was found. In a control experiment, a sample without CA prepared according to the same protocol was dissolved in water after a few hours (Figure A1).

**Figure 4:**
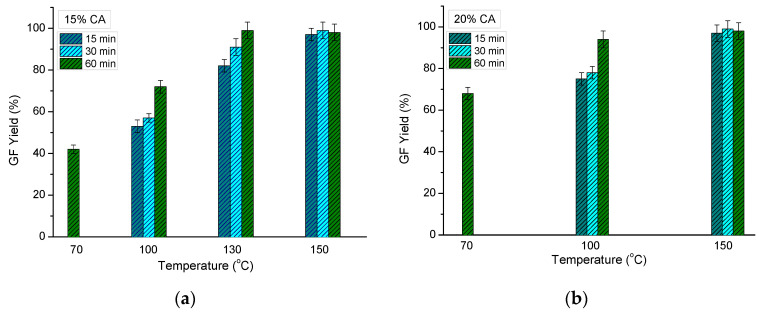
Effect of reaction temperature and reaction time on the gel fraction yield of HEC cryogels synthesized with 15 (**a**) and 20 mass % CA (**b**).

The influence of reaction temperature and time on the GF yield was evaluated as well (Figure 4). The heating of HEC samples, containing 15 and 20 mass % of CA (HEC/CA-15, HEC/CA-20), at 150 °C for 60 min resulted in a GF yield approaching the theoretical value of 100% (Figure 4). The decrease in reaction temperature gradually decreased the gel fraction of the polymer network. Hence, HEC crosslinked with 15% CA at 100 °C exhibited a rather low GF yield, while the crosslinking reaction at 70 °C took 60 min to yield gels with a gel fraction of only 42% (Figure 4a). Increasing the CA content from 15 to 20 mass % allowed us to obtain HEC cryogels with a relatively higher GF yield at 70 and 100 °C (Figure 4b).

**Figure A2:**
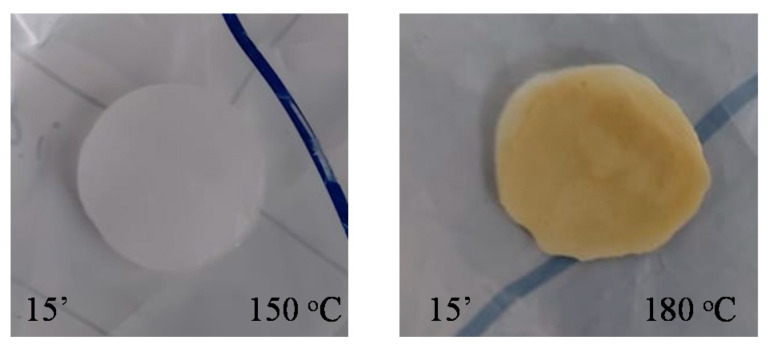
HEC/CA-15 cryogels crosslinked at 150 (**left**) and 180 °C (**right**) for 15 min.

A sample heated at a higher temperature (180 °C) changed its white color to dark yellow, which is associated with polymer degradation under the reported experimental conditions (Figure A2). The effect of reaction time on the GF yield was more pronounced at the lower reaction temperatures (Figure 4). As a rule, the longer the reaction time, the higher the GF yield. It should be mentioned that the GF yield values at 150 °C were very close and within the margin of error.

### 2.3. Swelling Degree

**Figure 5:**
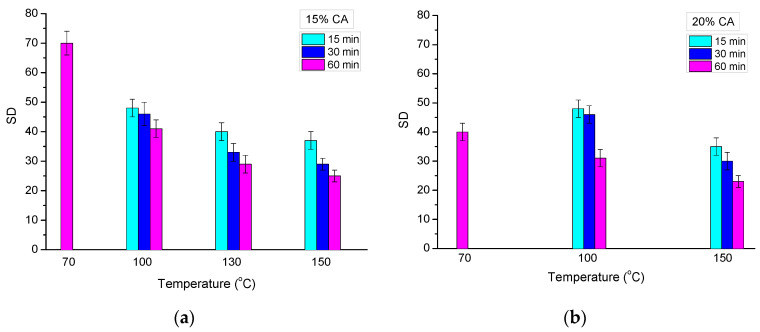
Effect of reaction temperature and reaction time on the swelling degree of HEC cryogels in water, synthesized with 15 (**a**) and 20 mass % CA (**b**).

The crosslinked dry disks were placed in water to afford HEC cryogels. Typically, for such materials, water was taken up for 1–2 sec, thanks to the interconnected pores. Nevertheless, the samples were allowed to swell in water for an additional 12 h, and then the swelling degree (SD) was determined. Noteworthily, all synthesized cryogels with a GF yield ≥ 50% remained intact, and only marginal change of their shape and size was observed (see Figure 2, bottom). The SD of HEC cryogels strongly depended on the two studied parameters of reaction time and reaction temperature (Figure 5). At the given temperature, the increase in reaction time resulted in decreased SD. On the other hand, samples heated for equal times at different temperatures exhibited a pronounced decrease in SD with the rise in temperature. Concerning the gels obtained at 70 °C (60 min), the SD of HEC/CA-15 was significantly higher than the SD of HEC/CA-20. At the higher reaction temperatures, the difference in SD was not so pronounced. These results are in line with the tendency observed for the GF yield.

### 2.4. Interior Morphology

**Figure 6:**
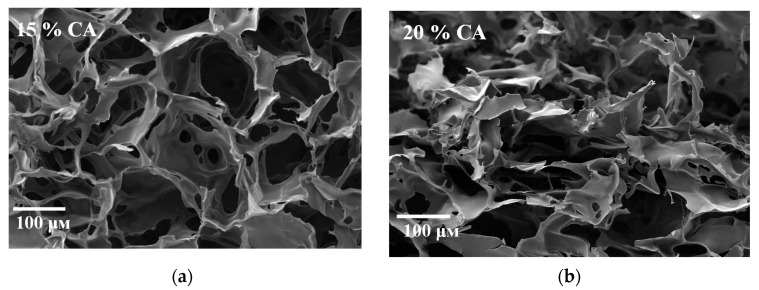
Scanning electron microscopy micrographs of freeze-dried HEC cryogels synthesized with 15 (**a**) and 20 mass % CA (**b**). Reaction time = 60 min; temperature = 150 °C.

The super-macroporous structure of HEC cryogels was visualized by scanning electron microscopy. Figure 6 presents the inner morphology of two selected freeze-dried samples, —HEC/CA-15 and HEC/CA-20. All studied gels possessed large, interconnected pores with dimensions between 100 and 180 μm. The pores were surrounded by thin, dense walls built by the polymer matrix. This spongy morphology imparts opacity to the cryogel materials.

### 2.5. Dynamic Rheological Properties

**Figure 7:**
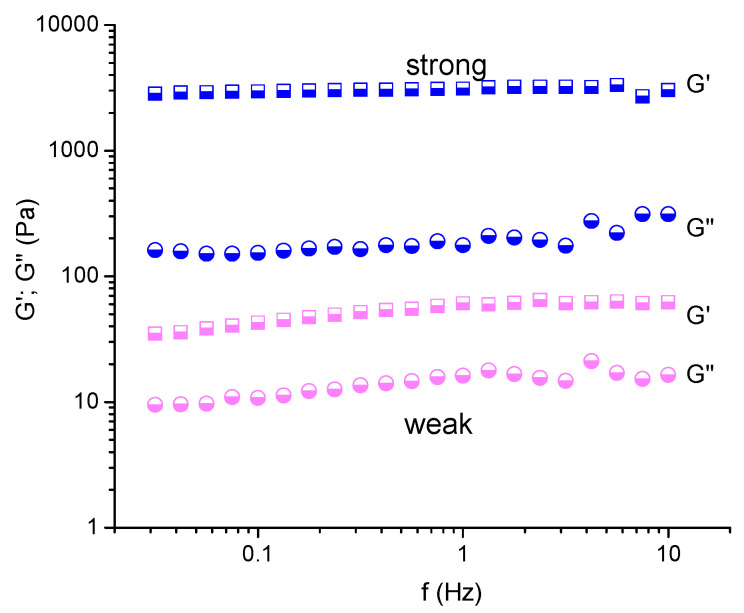
Variation of the elastic and loss moduli of HEC/CA-15 cryogels as a function of frequency. The weak and strong gels were obtained at 70 and 150 °C, respectively. Reaction time = 60 min.

The viscoelastic properties of HEC cryogels were studied using dynamic rheological analysis. This experiment was aimed at assessing the strength of materials obtained under different reaction conditions. In general, the storage (G’) and loss (G”) moduli of gels were nearly independent of the frequency. Figure 7 illustrates the variation of the G’ and G” of two cryogels, marked as weak and strong, in the 0.03–10 Hz frequency range. In both cases, the storage modulus was significantly higher than the loss modulus, telling us that the elastic response of gels to the applied shear stress considerably exceeds the viscous response.

**Figure 8:**
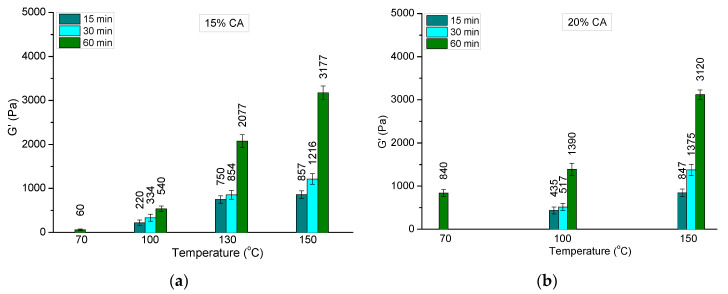
Effect of reaction temperature and reaction time on the elastic modulus of HEC cryogels, synthesized with 15 (**a**) and 20 mass % CA (**b**).

The reaction temperature has a tremendous effect on the elastic properties of HEC cryogels (Figure 8). Indeed, the G’ of the HEC/CA-15 cryogel, obtained at 150 °C (60 min), was 8 times higher than the G’ of HEC/CA-15 synthesized at 100 °C (60 min). A significant enhancement of the elastic modulus with increasing temperature was also found for the HEC/CA-20 series. In a similar way, G’ increased when the samples were heated for a longer time (at given temperature). As a rule, the reaction time of 60 min yielded HEC cryogels with much better elastic properties as compared to the materials crosslinked for 15 and 30 min.

### 2.6. Hydrolytic Degradation

**Figure 9:**
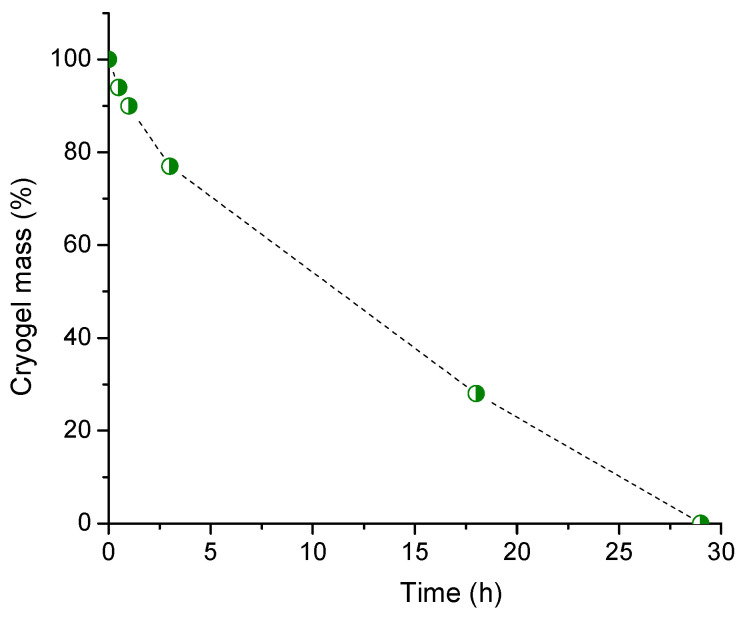
Mass loss of HEC/CA-20 cryogel during acid hydrolysis. The gel was synthesized by crosslinking HEC with CA for 15 min at 150 °C.

Simple acid hydrolysis was performed to give an idea of the degradability of CA-crosslinked HEC cryogels. HEC cryogel disks were placed in an HCl buffer (pH = 1.2) and kept at 80 °C for a given time. The tested sample was selected among the gels with very high GF yields. The mass loss and degradation of the cryogels occurred at a higher rate during the first few hours and then slowed down (Figure 9). The gel was completely degraded after 29 h.

## 3. Discussion

We introduced a novel strategy for fabricating green super-macroporous cryogel materials based on the chemical crosslinking of HEC with CA in a solid state. Cryogels were synthesized from two natural compounds, HEC and CA, without using any harmful chemical reagent or technology. Initially, HEC and CA were dissolved in water, and then the solution was frozen at −20 °C to create a macroporous structure. In this step, water played the role of solvent and structuring agent. Indeed, upon freezing, most of the water fraction formed interconnected ice crystals, while HEC and CA were accumulated in the so-called non-frozen liquid microphase, located between the crystals [9]. The aqueous phase was removed by freeze drying, and thus, a super-macroporous solid disk sample was obtained. We assume that this solid disk consisted of an HEC matrix in which CA molecules were homogeneously distributed. At an elevated temperature, CA tended to form anhydride intermediates, which then reacted with HEC hydroxyl groups to build the polymer network [15]. The reaction efficiency, estimated from the GF yield values, strongly depended on the reaction temperature and time, as well as the HEC/CA mass ratio. An HEC gel was obtained with a 5 mass % of CA; however, a very high GF yield (close to 100%) was reached when CA was ≥15 mass %. Obviously, at such concentration, almost all reagent molecules were incorporated into the polymer network. Highly efficient crosslinking occurred at temperatures equal to or higher than 100 °C (130 for samples containing 15% of CA) and a reaction time of 60 min. Moreover, the samples heated to 150 °C were converted into highly crosslinked gels for 10–15 min. The high degree of CA incorporation in the HEC network also affected the swelling ability and elastic properties of the material. The swelling degree varied between 17 and 40, depending on the experimental conditions. In fact, the longer reaction time and higher reaction temperature (estimated as better conditions for crosslinking) afforded gels with decreased SD and increased G’. In particular, the gels prepared at 150 °C for 60 min exhibited extremely good elastic properties. These results indicate the formation of a very dense polymer network. We should note that the storage modulus of HEC/CA-15 and HEC/CA-20 is approximately 20 times larger than the G’ of the photo-crosslinked pure HEC cryogel and 10 times larger than the G’ of HEC cryogels photo-crosslinked in the presence of 30 mass % of *N*,*N*’-methylenebisacrylamide [14]. Consequently, HEC cryogels with superior strengths can be obtained under the reported optimal reaction conditions. These gels can be used, for instance, as matrices to immobilize enzymes and cells, allowing for the repeated use of a material without compromising its integrity. On the other hand, the highly crosslinked HEC/CA-20 cryogel readily degraded in acidic conditions over 1 day. This relatively fast degradation process is probably due to the large, interconnected cryogel pores, which allowed unimpeded entry of acid molecules into the whole volume of the gel, and thus, the simultaneous cleavage of more glycosidic bonds, respectively.

## 4. Materials and Methods

### 4.1. Compounds and Reagents

2-Hydroxyethyl cellulose (1.3 KDa; degree of substitution 1.5) was donated by Hercules Inc. Aqualon Division (Wilmington, DE, USA). Citric acid (≥99.5%) was purchased from Sigma-Aldrich and used as received.

### 4.2. Synthesis of Cryogels

An appropriate amount of CA (0.05 g; 0.02 g; 0.03 g; 0.04 g) was dissolved in 10 mL of deionized water. The solution was heated with the aid of a magnetic stirrer at a temperature of 40 °C, and then 0.2 g of HEC was added slowly at a high stirring speed. Stirring was continued for 20 min. The samples were then allowed to stand at room temperature for 24 h to obtain a homogeneous solution. Eight portions of 1 mL of the solution were placed in Teflon molds, placed in a freezer, and kept at −20 °C for 2 h. The samples were freeze dried and placed in an oven and heated at different temperatures (70, 100, 130, and 150 °C).

### 4.3. Determination of Gel Fraction Yield and Swelling Degree

All samples were put in distilled water (100 mL) and purified by extraction at room temperature for 7 days. Water was exchanged four times. The gel fraction yield and swelling degree of the cryogels were determined gravimetrically using the following equations:

$$GF\left(\%\right)=\frac{\mathrm{mass}\;\mathrm{of}\;\mathrm{freeze}–\mathrm{dried}\;\mathrm{cryogel}}{\mathrm{initial}\;\mathrm{mass}\;\mathrm{of}\;\mathrm{HEC}\;\mathrm{and}\;\mathrm{CA}}\times 100$$

$$SD=\frac{\mathrm{mass}\;\mathrm{of}\;\mathrm{swollen}\;\mathrm{cryogel}}{\mathrm{mass}\;\mathrm{of}\;\mathrm{freeze}–\mathrm{dried}\;\mathrm{cryogel}}$$

### 4.4. Freeze Drying

HEC cryogels were freeze dried in an Alpha1–2 freeze dryer (Martin Christ) for 24 h at 0.02 mbar and −55 °C.

### 4.5. Rheological Measurements

Dynamic rheological measurements of HEC cryogels were conducted by using a HAAKE RheoStress 600 rheometer with a parallel plate sensor system (20 mm diameter) and Peltier temperature controller. Dynamic storage and loss moduli were measured in the 0.03–10 Hz frequency range in controlled deformation mode at γ = 0.005, which is inside the linear viscoelastic regime. Three runs of each sample were performed at 25 °C.

### 4.6. Scanning Electron Microscopy

Scanning electron microscopy analysis was performed by using a JEOL 6390 apparatus with an accelerating potential of 8.00 kV. All freeze-dried specimens were fractured and fixed on a glass substrate with nail polish and coated with gold for 60 s prior to taking measurements.

### 4.7. Hydrolytic Degradation

Hydrolytic degradation of HEC cryogels was carried out in an acidic medium (pH = 1.2). The acidic buffer was prepared by mixing 250 mL of an aqueous solution of KCl (0.2 M) and 425 mL of HCl (0.2 M) in a volumetric flask. The mixture was diluted with distilled water to 1L. The freeze-dried HEC cryogel disks were immersed in beakers filled with 100 mL of buffer solution and kept at 80 °C under gentle stirring (50 rpm). At a given time, disks were picked up from the buffer solution, washed with distilled water, freeze dried, and weighed. Mass loss (ML) was determined by the following equation:

$$ML\left(\%\right)=\frac{\mathrm{mass}\;\mathrm{of}\;\mathrm{acid}\;\mathrm{treated}\;\mathrm{freeze}–\mathrm{dried}\;\mathrm{cryogel}}{\mathrm{initial}\;\mathrm{mass}\;\mathrm{of}\;\mathrm{freeze}–\mathrm{dried}\;\mathrm{cryogel}}\times 100$$

## 5. Conclusions

Super-macroporous degradable cryogels were synthesized by a novel strategy involving thermally induced crosslinking of HEC with CA in a solid state. The crosslinking reaction was found to be efficient, and the GF yield was very high (close to 100%) with a CA concentration ≥ 15 mass % (with respect to HEC), a reaction temperature ≥ 100 °C (130 for samples containing 15% of CA), and a reaction time of 60 min. Moreover, the samples heated to 150 °C were transformed into highly crosslinked gels for only 10–15 min. The swelling degree of HEC cryogels varied between 17 and 40, depending on the experimental conditions. As a rule, longer reaction times and higher reaction temperatures afford gels with a decreased SD and an increased G’. HEC gels synthesized with CA as the crosslinking agent at 150 °C for 60 min were characterized by extremely good elastic properties, as their storage modulus exceeded approximately 20 times the G’ of the photo-crosslinked pure HEC cryogel and 10 times the G’ of HEC cryogels photo-crosslinked in the presence of 30 mass % *N*,*N’*-methylenebisacrylamide. Thus, the newly reported method allows for the fabrication of soft super-macroporous green materials based the chemical crosslinking of HEC with superior mechanical properties. We believe that this method is not restricted to the present system and can be extended to the fabrication of different cryogel systems, with potential for use in tissue engineering, pharmacology, biotechnology, etc.

## Data Availability

No data are available.

## Sample Availability

Samples of the compounds in this article are not available from the authors.
